# Supplementary material for: A Global Screen for Assembly State Changes of the Mitotic Proteome by SEC-SWATH-MS
Source: Cell Syst. 2020 Feb 26;10(2):133–155.e6. doi: 10.1016/j.cels.2020.01.001 (PMC7042714; doi:10.1016/j.cels.2020.01.001)

Q9BXW6 | OSBL1_HUMAN | OSBPL1A ORP1 OSBP8 OSBPL1 OSBPL1B
Monomer MW [kDa]: 108.47 Monomer expected elution fraction: 39

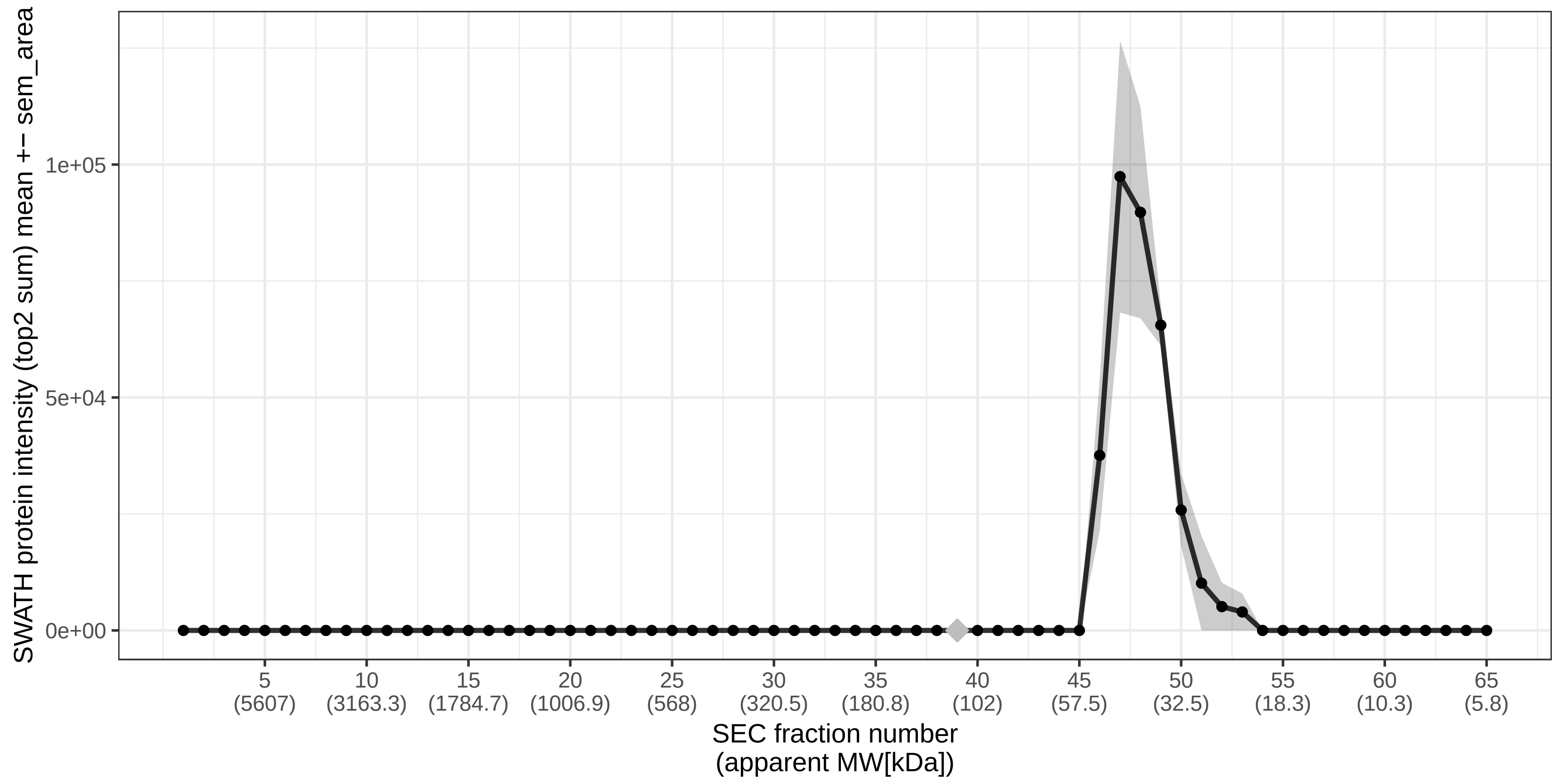

Supplement: Data S1. SEC-SWATH-MS Protein Chromatograms, Related to Figure 1 [file mmc6.zip › SECchrom_Q9BXW6_OSBL1_HUMAN_OSBPL1A_ORP1_OSBP8_OSBPL1_OSBPL.pdf]
